# Characterization of a new full length *TMPRSS3 *isoform and identification of mutant alleles responsible for nonsyndromic recessive deafness in Newfoundland and Pakistan

**DOI:** 10.1186/1471-2350-5-24

**Published:** 2004-09-24

**Authors:** Zubair M Ahmed, Xiaoyan Cindy Li, Shontell D Powell, Saima Riazuddin, Terry-Lynn Young, Khushnooda Ramzan, Zahoor Ahmad, Sandra Luscombe, Kiran Dhillon, Linda MacLaren, Barbara Ploplis, Lawrence I Shotland, Elizabeth Ives, Sheikh Riazuddin, Thomas B Friedman, Robert J Morell, Edward R Wilcox

**Affiliations:** 1 Section on Human Genetics, Laboratory of Molecular Genetics, National Institute on Deafness and Other Communication Disorders, National Institutes of Health, Rockville, MD, USA; 2 National Center of Excellence in Molecular Biology, Punjab University, Lahore, Pakistan; 3 Discipline of Genetics, Faculty of Medicine, Memorial University of Newfoundland, St. John's, NL, Canada; 4 Discipline of Pediatrics, Faculty of Medicine, Memorial University of Newfoundland, St. John's, NL, Canada; 5 Department of Genome Sciences, University of Washington, Seattle, WA, USA; 6 Department of Medical Genetics, Alberta Children's Hospital, Calgary, AB, Canada; 7 Hearing Section, National Institute on Deafness and Other Communication Disorders, National Institutes of Health, Bethesda, MD, USA; 8 Present Address: Section on Hereditary Disorders of the Ear, Laboratory of Molecular Oncology, Department of Cell and Molecular Biology, House Ear Institute, Los Angeles, CA, USA; 9 Present Address: James H. Quillen Veterans Affairs Medical Center, Mountain Home, TN, USA

## Abstract

**Background:**

Mutant alleles of *TMPRSS3 *are associated with nonsyndromic recessive deafness (DFNB8/B10). TMPRSS3 encodes a predicted secreted serine protease, although the deduced amino acid sequence has no signal peptide. In this study, we searched for mutant alleles of *TMPRSS3 *in families from Pakistan and Newfoundland with recessive deafness co-segregating with DFNB8/B10 linked haplotypes and also more thoroughly characterized the genomic structure of *TMPRSS3*.

**Methods:**

We enrolled families segregating recessive hearing loss from Pakistan and Newfoundland. Microsatellite markers flanking the TMPRSS3 locus were used for linkage analysis. DNA samples from participating individuals were sequenced for *TMPRSS3*. The structure of *TMPRSS3 *was characterized bioinformatically and experimentally by sequencing novel cDNA clones of *TMPRSS3*.

**Results:**

We identified mutations in *TMPRSS3 *in four Pakistani families with recessive, nonsyndromic congenital deafness. We also identified two recessive mutations, one of which is novel, of *TMPRSS3 *segregating in a six-generation extended family from Newfoundland. The spectrum of *TMPRSS3 *mutations is reviewed in the context of a genotype-phenotype correlation. Our study also revealed a longer isoform of *TMPRSS3 *with a hitherto unidentified exon encoding a signal peptide, which is expressed in several tissues.

**Conclusion:**

Mutations of *TMPRSS3 *contribute to hearing loss in many communities worldwide and account for 1.8% (8 of 449) of Pakistani families segregating congenital deafness as an autosomal recessive trait. The newly identified *TMPRSS3 *isoform *e *will be helpful in the functional characterization of the full length protein.

## Background

Genetic analysis of inherited deafness is a powerful tool for discovering the molecular mechanisms that control the development, function and maintenance of the auditory system. Linkage analysis of deafness segregating in large families has resulted in the mapping of more than 55 non-overlapping loci and the discovery of over 30 genes that are essential for hearing [1]. Given this extensive genetic heterogeneity, extended families from endogamous populations are ideally suited for identifying novel deafness genes and for genotype-phenotype studies.

DFNB8/B10, an autosomal recessive deafness locus, was independently mapped in two consanguineous families from Palestine and Pakistan [2,3]. Haplotype and gene sequence analyses of individuals in these two families led to the identification of mutations in a gene encoding a serine protease, TMPRSS3 [4,5]. TMPRSS3 belongs to a subfamily of type II transmembrane serine proteases, which also includes TMPRSS1, TMPRSS2, TMPRSS4 and TMPRSS5 [6]. The *TMPRSS3 *gene, spanning approximately 24 kb on chromosome 21, contains thirteen reported exons [4]. In humans there are alternatively spliced transcripts (*TMPRSS3 a*, *b*, *c *and *d*), encoding predicted polypeptides of 454, 327, 327 and 344 amino acids, respectively [4]. Here, we report the identification of a fifth isoform, *TMPRSS3e*, which encodes 538 amino acid residues, including a signal peptide, and is expressed in many human tissues.

We previously reported pathogenic *TMPRSS3 *mutations in four out of a total of 159 Pakistani families segregating profound congenital recessive deafness [5]. To determine the contribution of *TMPRSS3 *mutations to recessive deafness in Pakistan, we screened an additional 290 Pakistani families for linkage to the DFNB8/B10 locus. We also screened *TMPRSS3 *for mutations in a large kindred from Newfoundland, Canada segregating hearing loss linked to markers for DFNB8/B10 and surprisingly found two different mutant alleles.

## Methods

### Family ascertainment

Institutional review board approvals (OH93-N-016 and OH95-DC-N-050) were obtained for this study from the National Institutes of Health, USA, the Centre of Excellence in Molecular Biology, Lahore, Pakistan and the Newfoundland and Labrador Medical Genetics Program, Health Sciences Centre, St. John's. Participating individuals gave written informed consent. Medical histories indicated that all four Pakistani families segregate congenital, profound, non-syndromic sensorineural hearing loss. Pure tone air and bone conduction audiometry was performed on affected and unaffected individuals of the family from Newfoundland. Pneumatic otoscopy was used to confirm or deny cases in which conductive hearing loss was suspect. All testing was done in non-sound-attenuated exam room. Prior to testing, a biologic calibration was done in the room used for testing with a listener having normal hearing thresholds (LS). Possible effects of ambient noise on thresholds were then taken into account and only those frequencies unaffected were included. DNA was extracted from venous blood samples from the participating individuals.

### Linkage and sequence analyses

DNA samples were PCR amplified using fluorescently labeled primers surrounding microsatellite repeats at known DFNB loci and analyzed on an ABI 377 DNA sequencer. Genotypes were determined using Genescan and Genotyper software (PE Biosystems). To detect *TMPRSS3 *mutations in families segregating DFNB8/B10, the sequence of each exon of *TMPRSS3 *was evaluated in two affected subjects from each family as described previously [5].

### 5' RACE and cloning of *TMPRSS3e*

Sequence analysis of the subcloned PCR product amplified from human retina GETLarge full length cDNA (Genemed Biotechnologies) using primers 5'GGGTTGCTTCAAATGGCTTACTAGATCC3' and 5'CATTTTCCCCCATGGTGACTATTTCAG revealed an additional 385 bp of transcribed sequence downstream of *TMPRSS3a *exon 1. 5' RACE PCR using human retina Marathon-Ready cDNA as template (Clontech) with primers 5'CAGACCAATGGCCAGTGCTAATATC3' and the AP1 primer was performed using the following thermal cycling conditions: 94°C for 1 min, 25 cycles of 94°C for 30 s and 72°C for 5 min. Five microliters of amplified PCR product was used for the second round of amplification with nested primer 5'TTTTCAAATCATCAAGGCCAAAAAG3' and the AP2 primer. Five microliters of each sample were analyzed on a 1.2% agarose/ethidium bromide gel, extracted using a QIAquick gel extraction kit (Qiagen) and cloned into *E. coli *using pGEM^®^-T Easy vector (Promega). DNA was purified from minipreps using QIAquick miniprep kit (Qiagen) and inserts were sequenced using T7 and SP6 primers. To amplify the full length *TMPRSS3e *isoform, 5'ATGGTGAGTAAAATGGGTGTGAGGA3' and 5'CTTGGAAGTAGAAAGGGTGGGTTTG3' primers and LA-Taq polymerase (Pan Vera) were used as recommended by the manufacturers.

### Multiple-tissue cDNA panel analysis

Expression of *TMPRSS3e *was evaluated using a Human Tissue cDNA panel (Clontech). *TMPRSS3e *cDNA was amplified with primers 5'CCAGAAATGGTGAGTAAAATGG3' and 5'AGCAACAGCATCTGCATCTGGT3' using 20 pg of each cDNA as template following the manufacturer's recommended protocol (Clontech). PCR amplified products were sub-cloned into pGEM-T Easy vector (Promega) and sequenced.

## Results

**Figure 2:**
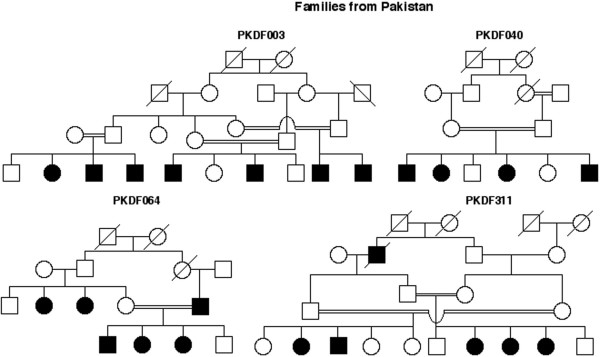
**Pedigrees of Pakistani families. **Four families with nonsyndromic recessive deafness from Pakistan.

In four out of 290 newly investigated Pakistani families, nonsyndromic congenital deafness was found to co-segregate with DFNB8/B10 linked haplotypes (Figure 2). Mutational analysis of DNA samples from the affected individuals from these families (Table 1) revealed three previously reported mutations (207delC, C407R, C194F). In our families segregating 207delC there is a common disease associated haplotype [5]. The second mutation, C407R, was found previously in affected individuals of two Pakistani families and in a heterozygous state in one person of Indian descent. The same C407R ancestral haplotype was also found in family PKDF040 [5]. The third mutation, C194F, was reported in one Pakistani family [5], which has the same haplotype as family PKDF064 (Figure 2).

**Table 1:** Mutant alleles of *TMPRSS3 *found in this study

Family	Mutation	Domain	Amino acid substitution	First reference
Family B	IVS8+8insT	SP	Frameshift	This study
Family B	207delC	LDLRA	Frameshift	[9]
PKDF003	207delC	LDLRA	Frameshift	[9]
PKDF040	1219T>C	SP	C407R	[5]
PKDF064	581G>T	SRCR	C194F	[5]
PKDF311	207delC	LDLRA	Frameshift	[9]

SP; Serine Protease, LDLRA; Low Density Lipoprotein Receptor A, SRCR; Scavenger-Receptor Cysteine-Rich

**Figure 1:**
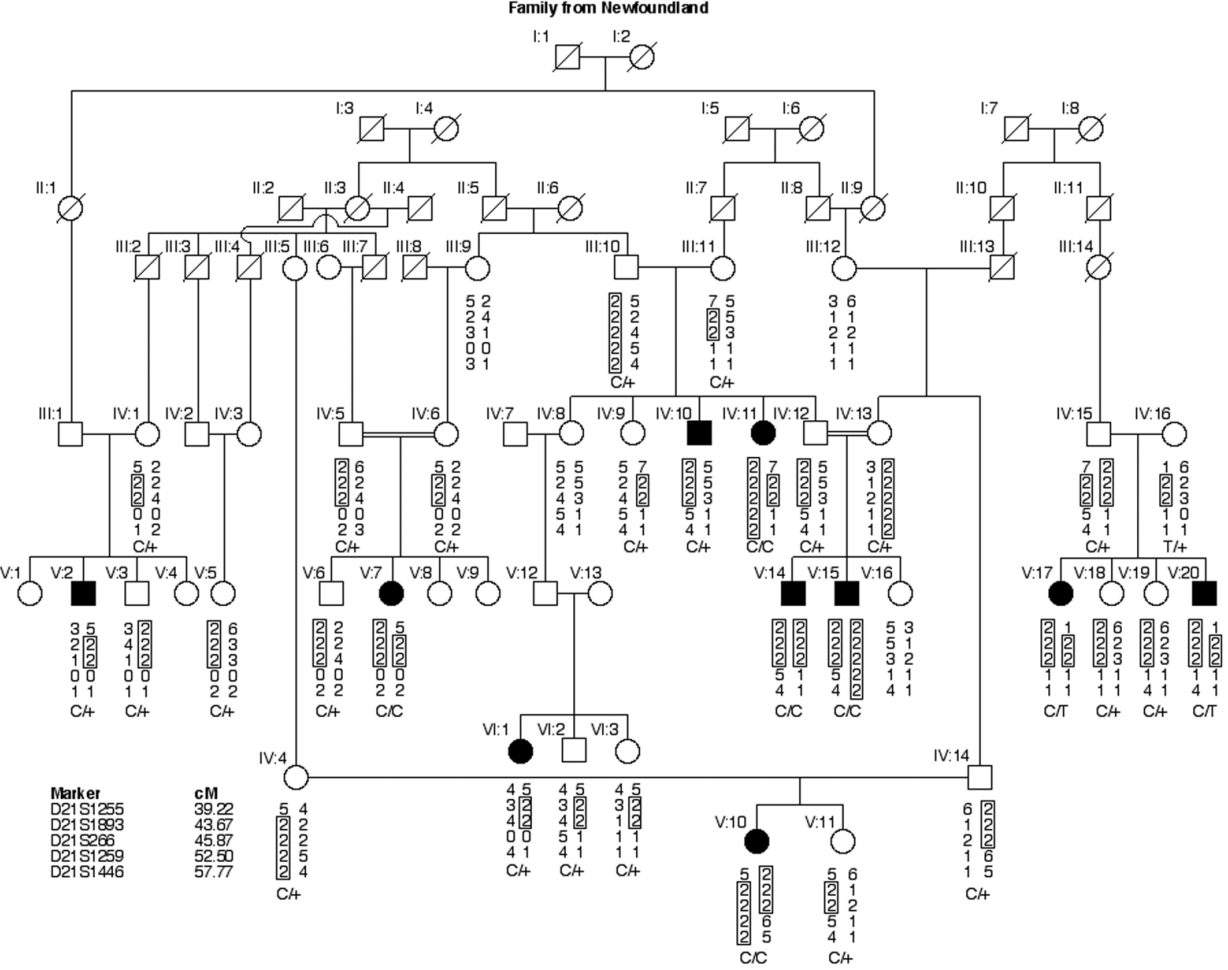
**Pedigree of Newfoundland family. **There are ten hearing impaired individuals in a six generation extended family structure. Drawn below the enrolled subjects is a haplotype for STR markers around the DFNB8/B10 locus on chromosome 21. The carrier status of each person for the mutant alleles of *TMPRSS3 *found in this family is shown. "C" represents 207delC, while "T" stands for IVS8+8insT. Individuals V:17 and V:20 are compound heterozygotes.

After completing a genome wide screen we found linkage of deafness to markers for DFNB8/B10 in one branch of a large six-generation pedigree (Figure 1) from Labrador [7], a Canadian island whose small population lives in villages isolated by geography and weather [8]. Medical histories and pure tone air and bone conduction audiometry revealed nonsyndromic, pre-lingual, severe to profound, sensorineural hearing impairment in ten participating affected individuals. Linkage analysis in this family demonstrated homozygosity for DFNB8/B10 linked microsatellite markers among seven affected individuals from five different sibships (Figure 1). An apparent founder DFNB8/B10 associated haplotype was constructed using the available genetic information (Figure 1). Three hearing impaired individuals (IV:10, V:2 and VI:1) carry only a single copy of the DFNB8/B10 haplotype, although their deafness is clinically indistinguishable from that of the five homozygotes.

Sequencing of the *TMPRSS3 *gene in affected individuals revealed two mutant alleles. The first mutation occurs in exon 4 of *TMPRSS3 *and is a deletion of a cytosine at position 207 (207delC) of the mRNA (Figure 1, Table 1). The second mutation inserts a "T" residue at the eighth position after the splice donor site of exon 8 (IVS8+8insT). Although the precise effect of this mutant allele is not known, Genscan predicts this results in the skipping of exon nine. Individual IV:16 is a carrier of IVS8+8insT and her two affected children are compound heterozygotes (Figure 1). Neither of these two mutations was found in 100 random normal control DNA samples (200 chromosomes) from Newfoundland.

**Figure 3:**
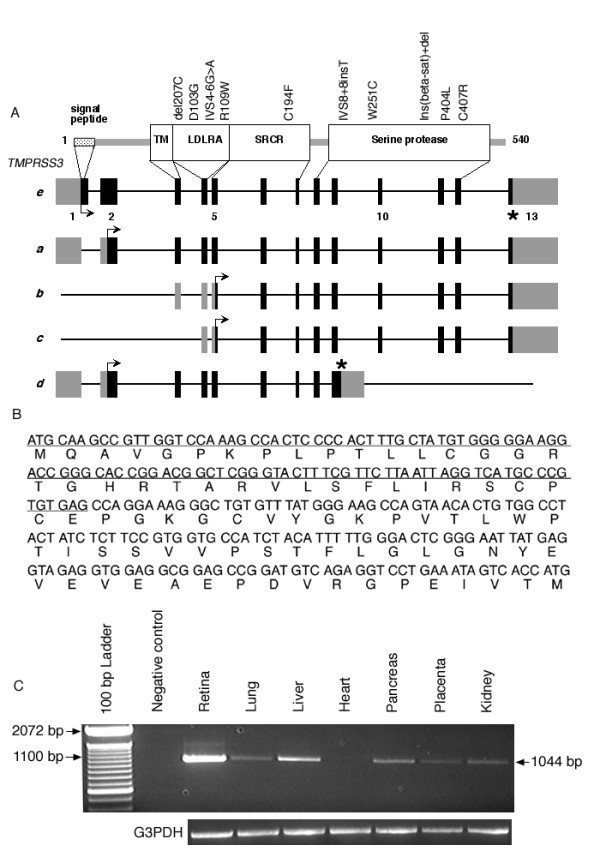
**Mutational spectrum, structure and expression of *TMPRSS3 *isoforms. **(A) Coding and non-coding exons of all the known isoforms of *TMPRSS3 *are shown with black and gray rectangles, respectively. The newly identified isoform *e *has translation initiation codon (arrow) in exon 1, while the termination codon in exon 13 is marked with an asterisk. Shown also are predicted protein motifs encoded by the 3192 bp long mRNA of isoform *e*. All the known mutant alleles of *TMPRSS3 *causing hearing loss are shown above the protein motifs. Modified and updated from Ben-Yosef et al. 2001 [5] (B) Nucleotide sequence of the cDNA encoding the amino terminus of *TMPRSS3e *and its deduced amino acid sequence. The underlined nucleotide sequence represents the region predicted by SMART to encode a signal peptide. The last ATG shown is the reported translation initiation site for isoform *a *[4]. (C) RT-PCR specific to the *TMPRSS3e *transcript was performed on cDNA from seven human tissues, which include retina, lung, liver, heart, pancreas, placenta and kidney as indicated. All tissues, except heart, demonstrated expression of *TMPRSS3e*. *G3PDH *was used as a positive control.

We previously reported a Pakistani family (PKSR7) in which deaf subjects were homozygous for the markers spanning the DFNB8/B10 region with the simulated maximal lod score of 3.8 [5]. However, we did not find a mutation in the known coding and non-coding exons of *TMPRSS3 *[5]. One possible explanation is that family PKSR7 has a mutation in a regulatory element of *TMPRSS3*. Alternatively, there might be additional uncharacterized exons of *TMPRSS3*. Considering the later possibility, we searched for additional exons and alternatively spliced transcripts of *TMPRSS3*. Previous studies have documented four isoforms of *TMPRSS3 *[4]. To probe for novel coding sequence of *TMPRSS3 *we designed primers from the known exons to amplify smaller overlapping cDNA product(s). The amplicon generated using a forward primer from the reported non-coding exon 1 and a reverse primer from the protein coding region of exon 2 was larger than the expected size product based on the sequence of *TMPRSS3 *isoform *a *[4]. After subcloning and sequencing this transcript, an additional 385 bp of sequence was found at the 3' end of exon 1 [4]. We further confirmed the results by 5'RACE using gene specific primers and human Marathon-Ready retina cDNA as template. Using specific primers we amplified a full-length transcript of this novel isoform, which we designated *TMPRSS3e *(Figure 3).

Although the ATG codon at position 705 (Accession # AY633572) does not meet Kozak consensus criteria, there are two reasons for believing that this is the translation initiation site. First, there are stop codons in all three reading frames upstream of this site. Secondly, the 252 bp open reading frame between ATG 705 and ATG 957 (Figure 3A), the previously reported initiation codon, includes a predicted signal peptide, which is expected in a secreted serine protease. Considering the ATG at position 705 as a starting codon, analysis of the 1614 bp open reading frame indicates that this larger *TMPRSS3e *transcript encodes a putative polypeptide of 538 amino acids, including 84 previously unknown amino acids at the amino terminus (Figure 3B and 3C). The SMART program predicts a cleavable signal sequence between residues 1 through 36 (Figure 3A and 3B). Among all the known isoforms of *TMPRSS3*, only isoform *e *reported here has a predicted signal peptide, consistent with the hypothesis that *TMPRSS3 *encodes a secreted serine protease. The remaining 48 new amino acids do not contain other predicted structural or functional motifs (Figure 3A and 3B). Downstream of the newly identified 84 amino acids, isoform e shares a common protein sequence with isoform a, which includes a transmembrane (TM), low density lipoprotein receptor A (LDLRA), scavenger-receptor cysteine-rich (SRCR) and serine protease domains (Figure 3A). Through non-quantitative RT-PCR analysis with *TMPRSS3e *specific primers and multiple human cDNAs, we found expression of this isoform in retina, lung, liver, pancreas, placenta and kidney (Figure 3C). Mutational screening of exon 1 and exon 2 as well as other exons of *TMPRSS3e*, using DNA samples from two affected individuals of family PKSR7 did not reveal a pathogenic change. This may be an indication of as yet uncharacterized additional exons of *TMPRSS3*, or the hearing loss phenotype in this family is due to a mutation in a regulatory element of *TMPRSS3*. Alternatively, it remains possible that family PKSR7 has a mutation in a gene closely linked to *TMPRSS3*.

## Discussion

*TMPRSS3 *is the only protease reported thus far to be involved in nonsyndromic deafness [4]. In this study, we document the mutant alleles of *TMPRSS3 *segregating in a large family from Newfoundland and in additional Pakistani families with nonsyndromic, recessive deafness. The Newfoundland family has at least two different mutated genes associated with hearing impairment. The majority of the affected individuals in this family are homozygous for 207delC (Figure 1), while two of the hearing impaired subjects are compound heterozygotes for 207delC and a newly described mutation of intron 8 (IVS8+8insT). The 207delC mutation appears to be wide-spread; it has been found in affected individuals of Spanish (one homozygote), Greek (one heterozygote), Newfoundland (homozygous individuals) and Pakistani (homozygous individuals, PKDF003 and PKDF311) origins, and is the most common of the mutant alleles of *TMPRSS3*. Although there is a predicted effect of IVS8+8insT mutation on splicing of exon 9, further studies are required to determine the precise role of IVS8+8insT on *TMPRSS3 *transcripts. The family from Pakistan that originally defined the DFNB8 locus was reported to have childhood onset hearing impairment [3], while all remaining families segregating *TMPRSS3 *mutations have pre-lingual deafness [4,5,9,10]. Mutational screening revealed a splice site mutation (IVS4-6G>A) in the original DFNB8 family, which may allow some normal splicing of *TMPRSS3 *transcripts [4]. The second mutant allele (IVS8+8insT) in the Newfoundland family may also be a leaky mutation, although the two individuals who are compound heterozygotes were described as having prelingual hearing impairment.

The hearing impaired individuals IV:10, V:2 and VI:1 of our Newfoundland family are carriers of 207delC and we did not find any other *TMPRSS3 *mutation in these individuals. Individuals IV:10 (affected) and IV:12 (normal hearing sibling) show an identical chromosome 21 haplotype. Similarly the individuals VI:1 (affected) and VI:2 (normal hearing sibling) share identical haplotypes for the *TMPRSS3 *locus. Hence, it is unlikely that the deafness of individuals IV:10 and VI:1 is due to cryptic *TMPRSS3 *mutations (Figure 1). As connexin 26 mutations are the most common cause of nonsyndromic recessive deafness [11], we sequenced the coding exon of *GJB2 *in affected individuals of Newfoundland family and found no mutation.

We have analyzed a total of 449 Pakistani families [[5] and this study] segregating severe to profound congenital recessive deafness and found a total of eight families in which the deafness phenotype is due to mutations of *TMPRSS3*. Therefore, the relative contribution of *TMPRSS3 *mutations in the deaf Pakistani population is approximately 1.8%, a significant amount considering the extensive genetic heterogeneity of deafness in this population. Of ten *TMPRSS3 *mutations that have been reported world wide, five (IVS4-6G>A, 207delC, R109W, C194F and C407R) are segregating in the Pakistani population [4,5,9,10].

Among the ten known mutant alleles of *TMPRSS3*, six are present in exons common to all isoforms, including isoform "*e*" reported herein. Of the isoforms of this serine protease, *TMPRSS3e *has the longest open reading frame and is the only isoform of this gene with a predicted signal sequence at the amino terminus. The identification of 84 additional amino acids, including a predicted signal sequence, may aid functional assays to determine the role of *TMPRSS3 *in cochlear development and function. *TMPRSS3 *isoform *a *was identified with a forward primer (TMa-F; see supplement table B [4]) in exon 2 and not in exon 1 as illustrated in figure 1 of Scott et al. 2001 [4]. Therefore, it is possible that *TMPRSS3a *is an incomplete version of isoform *e *and isoform *a *may not exist *in vivo*.

*TMPRSS3 *message is expressed in supporting cells of the organ of Corti, in the stria vascularis and in the spiral ganglion cells of the cochlea [12]. Although the specific role of *TMPRSS3 *in the development and maintenance of the audiosensory apparatus is still unknown, the reported mutant alleles of TMPRSS3 abolish catalytic activity of the serine protease, implying a proteolytic function during the inner ear development [12,13]. Although the *in vivo *substrate(s) of *TMPRSS3 *have not been reported in the auditory system, TMPRSS3 is thought to regulate the activity of the epithelial amiloride sensitive sodium channel (ENaC) *in vitro*, which was suggested to control critical signaling pathway(s) in the inner ear and may have a role in the maintenance of the low sodium concentration of endolymph [12]. However, the absence of an abnormal auditory phenotype in individuals with Pseudohypoaldosteronism type I (PHA 1), which are homozygous for null alleles of ENaC subunits [14], suggests that the DFNB8/B10 deafness phenotype is due to aberrant proteolytic processing by TMPRSS3 of some other substrate in the inner ear.

## Conclusions

*TMPRSS3 *mutations account for hearing loss in 1.8% (8 of 449) of Pakistani families segregating deafness as an autosomal recessive trait. We also identified two recessive mutations of *TMPRSS3 *segregating in a six-generation extended family from Newfoundland. Our study also revealed a longer isoform of *TMPRSS3 *with an exon encoding a signal peptide, which should help in the functional dissection of this secreted serine protease.

## Abbreviations

TMPRSS3; Transmembrane Protease, Serine 3 [OMIM entry 605511].

DFNB8; Deafness, childhood-onset neurosensory autosomal recessive 8 [OMIM entry 601072]DFNB10; Deafness, congenital neurosensory, autosomal recessive 10 [OMIM entry 605316]

## Competing interests

The authors declare that they have no competing interests.

## Authors' contributions

ZMA performed DNA sequencing, characterized the novel *TMPRSS3 *isoform *e *and prepared the manuscript. XCL analyzed all linkage data from family B from Newfoundland and identified DFNB8/B10 segregation in this family. SDP performed DNA sequencing to determine mutant alleles of TMPRSS3 and confirmed linkage in Pakistani families. SR, KR and ZA enrolled families in Pakistan. TLY re-sampled some members of family B from Newfoundland, confirmed genotypes and sequenced normal controls from Newfoundland. SL participated in the clinical assessment of Newfoundland deaf family members. KD sequenced normal controls from Newfoundland. LM was the genetic counsellor involved in family B enrolment from Newfoundland. BP provided considerable technical assistance. LIS performed hearing tests on Newfoundland family members. EI identified Newfoundland families with inherited deafness. SR was responsible for the enrolment of 449 families from Pakistan. TBF was in charge of oversight, editing and analyses. RJM and ERW trained investigators and were involved in the experimental design and genetic analyses of all families. All authors contributed to and edited the manuscript.

## Pre-publication history

The pre-publication history for this paper can be accessed here:



## References

[B1] Friedman TB, Griffith AJ (2003). Human nonsyndromic sensorineural deafness. Annu Rev Genomics Hum Genet.

[B2] Bonne-Tamir B, DeStefano AL, Briggs CE, Adair R, Franklyn B, Weiss S, Korostishevsky M, Frydman M, Baldwin CT, Farrer LA (1996). Linkage of congenital recessive deafness (gene DFNB10) to chromosome 21q22.3. Am J Hum Genet.

[B3] Veske A, Oehlmann R, Younus F, Mohyuddin A, Muller-Myhsok B, Mehdi SQ, Gal A (1996). Autosomal recessive non-syndromic deafness locus (DFNB8) maps on chromosome 21q22 in a large consanguineous kindred from Pakistan. Hum Mol Genet.

[B4] Scott HS, Kudoh J, Wattenhofer M, Shibuya K, Berry A, Chrast R, Guipponi M, Wang J, Kawasaki K, Asakawa S, Minoshima S, Younus F, Mehdi SQ, Radhakrishna U, Papasavvas MP, Gehrig C, Rossier C, Korostishevsky M, Gal A, Shimizu N, Bonne-Tamir B, Antonarakis SE (2001). Insertion of beta-satellite repeats identifies a transmembrane protease causing both congenital and childhood onset autosomal recessive deafness. Nat Genet.

[B5] Ben-Yosef T, Wattenhofer M, Riazuddin S, Ahmed ZM, Scott HS, Kudoh J, Shibuya K, Antonarakis SE, Bonne-Tamir B, Radhakrishna U, Naz S, Ahmed Z, Pandya A, Nance WE, Wilcox ER, Friedman TB, Morell RJ (2001). Novel mutations of TMPRSS3 in four DFNB8/B10 families segregating congenital autosomal recessive deafness. J Med Genet.

[B6] Szabo R, Wu Q, Dickson RB, Netzel-Arnett S, Antalis TM, Bugge TH (2003). Type II transmembrane serine proteases. Thromb Haemost.

[B7] Ives E, Collis E (1991). Severe childhood deafness on Newfoundland's south coast. Am J Hum Genet.

[B8] Rahman P, Jones A, Curtis J, Bartlett S, Peddle L, Fernandez BA, Freimer NB (2003). The Newfoundland population: a unique resource for genetic investigation of complex diseases. Hum Mol Genet.

[B9] Wattenhofer M, Di Iorio MV, Rabionet R, Dougherty L, Pampanos A, Schwede T, Montserrat-Sentis B, Arbones ML, Iliades T, Pasquadibisceglie A, D'Amelio M, Alwan S, Rossier C, Dahl HH, Petersen MB, Estivill X, Gasparini P, Scott HS, Antonarakis SE (2002). Mutations in the TMPRSS3 gene are a rare cause of childhood nonsyndromic deafness in Caucasian patients. J Mol Med.

[B10] Masmoudi S, Antonarakis SE, Schwede T, Ghorbel AM, Gratri M, Pappasavas MP, Drira M, Elgaied-Boulila A, Wattenhofer M, Rossier C, Scott HS, Ayadi H, Guipponi M (2001). Novel missense mutations of TMPRSS3 in two consanguineous Tunisian families with non-syndromic autosomal recessive deafness. Hum Mutat.

[B11] Cryns K, Orzan E, Murgia A, Huygen PL, Moreno F, del Castillo I, Chamberlin GP, Azaiez H, Prasad S, Cucci RA, Leonardi E, Snoeckx RL, Govaerts PJ, Van de Heyning PH, Van de Heyning CM, Smith RJ, Van Camp G (2004). A genotype-phenotype correlation for GJB2 (connexin 26) deafness. J Med Genet.

[B12] Guipponi M, Vuagniaux G, Wattenhofer M, Shibuya K, Vazquez M, Dougherty L, Scamuffa N, Guida E, Okui M, Rossier C, Hancock M, Buchet K, Reymond A, Hummler E, Marzella PL, Kudoh J, Shimizu N, Scott HS, Antonarakis SE, Rossier BC (2002). The transmembrane serine protease (TMPRSS3) mutated in deafness DFNB8/10 activates the epithelial sodium channel (ENaC) in vitro. Hum Mol Genet.

[B13] Lee YJ, Park D, Kim SY, Park WJ (2003). Pathogenic mutations but not polymorphisms in congenital and childhood onset autosomal recessive deafness disrupt the proteolytic activity of TMPRSS3. J Med Genet.

[B14] Chang SS, Grunder S, Hanukoglu A, Rosler A, Mathew PM, Hanukoglu I, Schild L, Lu Y, Shimkets RA, Nelson-Williams C, Rossier BC, Lifton RP (1996). Mutations in subunits of the epithelial sodium channel cause salt wasting with hyperkalaemic acidosis, pseudohypoaldosteronism type 1. Nat Genet.

